# The Role of Inorganic Nanomaterials in Overcoming Challenges in Colorectal Cancer Diagnosis and Therapy

**DOI:** 10.3390/pharmaceutics17040409

**Published:** 2025-03-25

**Authors:** Jun Wang, Hanwenchen Wang, Falong Zou, Junnan Gu, Shenghe Deng, Yinghao Cao, Kailin Cai

**Affiliations:** 1 Department of Gastrointestinal Surgery, Union Hospital, Tongji Medical College, Huazhong University of Science and Technology, Wuhan 430022, China; wj9711med@hust.edu.cn (J.W.); m202476342@hust.edu.cn (H.W.); m202376154@hust.edu.cn (F.Z.); 2 Department of Thoracic Surgery, Union Hospital, Tongji Medical College, Huazhong University of Science and Technology, Wuhan 430022, China; gujunnan@hust.edu.cn; 3 Center for Liver Transplantation, Union Hospital, Tongji Medical College, Huazhong University of Science and Technology, Wuhan 430022, China; dengshenghe@hust.edu.cn; 4 Cancer Center, Union Hospital, Tongji Medical College, Huazhong University of Science and Technology, Wuhan 430022, China; 5 Departments of Diagnostic Radiology, Surgery, Chemical and Biomolecular Engineering, and Biomedical Engineering, Yong Loo Lin School of Medicine and College of Design and Engineering, National University of Singapore, Singapore 119074, Singapore; 6 Clinical Imaging Research Centre, Centre for Translational Medicine, Yong Loo Lin School of Medicine, National University of Singapore, Singapore 117599, Singapore

**Keywords:** inorganic nanomaterials, colorectal cancer, diagnosis, drug delivery, treatment

## Abstract

Colorectal cancer poses a significant threat to human health due to its high aggressiveness and poor prognosis. Key factors impacting patient outcomes include post-surgical recurrence, chemotherapeutic drug resistance, and insensitivity to immunotherapy. Consequently, early diagnosis and the development of effective targeted therapies are essential for improving prevention and treatment strategies. Inorganic nanomaterials have gained prominence in the diagnosis and treatment of colorectal cancer owing to their unique size, advantageous properties, and high modifiability. Various types of inorganic nanomaterials—such as metal-based, metal oxide, quantum dots, magnetic nanoparticles, carbon-based, and rare-earth nanomaterials—have demonstrated significant potential in enhancing multimodal imaging, drug delivery, and synergistic therapies. These advancements underscore their critical role in improving therapeutic outcomes. This review highlights the properties and development of inorganic nanomaterials, summarizes their recent applications and progress in colorectal cancer diagnosis and treatment, and discusses the challenges in translating these materials into clinical use. It aims to provide valuable insights for future research and the clinical application of inorganic nanomaterials in colorectal cancer management.

## 1. Introduction

Colorectal cancer ranks as the third most prevalent and second most deadly cancer worldwide [1]. According to the latest statistics from the American Cancer Society, it has become the leading cause of cancer-related deaths among men in the United States, with 152,810 new cases anticipated in 2024 [2]. These alarming morbidity and mortality rates underscore the urgency of optimizing diagnostic and therapeutic approaches for colorectal cancer. Current international guidelines recommend endoscopic or surgical resection for early-stage colorectal cancer [3,4]. For advanced cases, a combination of radiotherapy and immunotherapy is often advocated to inhibit recurrence and metastasis [5,6]. However, challenges such as postoperative recurrence, the high costs of radiotherapy, the development of tumor resistance, low targeting efficiency, poor bioavailability, and significant side effects limit the accessibility and effectiveness of current treatment [7]. Achieving early diagnosis and developing more efficient, precise therapies with reduced toxicity have become critical goals in colorectal cancer management. In recent years, nanomedicine has emerged as a transformative tool in the diagnosis and treatment of various diseases. Nanomaterials, with their precise size control, exceptional drug delivery capabilities, and optical and magnetic properties, have shown considerable promise in oncology [8,9]. These materials exhibit unique targeting abilities, including passive targeting via the enhanced permeability and retention (EPR) effect to evade the human reticuloendothelial system (RES) [10,11,12] and active targeting achieved through surface modification [13,14,15]. While earlier research in cancer therapy focused primarily on organic nanomaterials such as liposomes, recent advancements have shifted attention toward inorganic nanomaterials (INPs) due to their superior physicochemical properties [16]. INPs demonstrate unique attributes, such as high photosensitivity, electrical conductivity, magnetic induction, and thermal conversion efficiency [17,18,19]. These properties allow INPs to function as both drug delivery platforms and therapeutic agents. INPs are derived from a variety of metals, metal oxides, and non-metallic materials (e.g., carbon, silicon dioxide, and rare-earth elements), offering excellent drug-loading capacity, as well as photodynamic therapy (PDT), photothermal therapy (PTT), and magnetic capabilities, which provide significant advantages in cancer therapy [20,21,22]. Furthermore, INPs enable advanced imaging techniques such as computed tomography (CT), magnetic resonance imaging (MRI), and photoacoustic imaging [23,24,25,26], enhancing tumor diagnosis and facilitating drug release modulation. Their nanoscale size also supports passive tumor targeting through mechanisms like the EPR effect [27,28] and active targeting via surface modifications [29,30]. These multifunctional advantages make INPs highly promising candidates for advanced colorectal cancer therapy, offering improvements over traditional radiotherapy drugs in terms of precision, efficacy, and reduced side effects.

There is a substantial body of literature on the use of INPs in drug delivery, diagnosis, and treatment of colorectal cancer [31,32]. However, their clinical application remains limited, with only two INPs currently approved for cancer therapy. The first approved INP was Fe_3_O_4_ nanoparticles (marketed as NanoTherm^®^ by MagForce Nanotechnologies AG (Berlin, Germany)), which received European Medicines Agency (EMA) approval in 2010 for the treatment of glioblastoma via thermal ablation using a magnetic field [33]. The second was hafnium oxide nanoparticles (marketed as Hensify^®^ [NBTXR3] by Nanobiotix (Paris, France)), approved in 2019 for treating locally advanced soft tissue sarcomas [34]. In recent years, advancements in nanomedicine have led to an increasing number of INPs entering clinical trials. Table 1 provides details on both past and ongoing clinical trials. For instance, Sichuan Enray Pharmaceutical Sciences Company (Chengdu, China) is conducting a phase I clinical trial to assess the potential efficacy of carbon nanoparticle-loaded iron [CNSI-Fe (II)] in advanced solid tumors, including colorectal and pancreatic cancers (NCT06048367) [35,36,37]. Similarly, NanoEcho AB (Uppsala, Sweden) is conducting a phase II clinical trial using ultrasmall superparamagnetic iron oxide (USPIO)-based contrast agent for rectal cancer (NCT06693375) [38,39]. This trial aims to improve the evaluation of lymph node metastasis and staging in rectal cancer patients, with completion expected by 2026. Accelerating the safe and efficient translation of inorganic nanomaterials from laboratory research to clinical practice has become a critical challenge in the development of nanomedicine. This paper reviews the classification and characterization of INPs, highlights their recent applications and progress in colorectal cancer research, discusses major obstacles and potential solutions for their clinical translation, and offers valuable insights for advancing nanomedicine in colorectal cancer.

## 2. Classification and Characterization of Inorganic Nanomaterials

**Figure 1:**
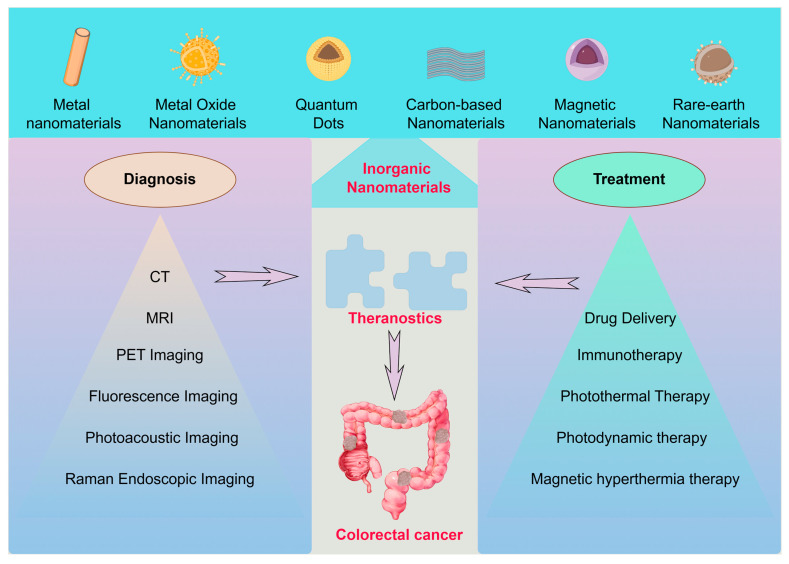
Classification of INPs in the diagnosis and treatment of colorectal cancer.

INPs are nanoscale materials composed of inorganic compounds, distinguished by their unique physical and chemical properties. These properties enable a wide range of applications across fields such as biomedicine, electronics, and catalysis. Based on their composition, structure, and applications, INPs can be broadly categorized into metal nanomaterials, metal oxide nanomaterials, quantum dots, magnetic nanomaterials, carbon-based nanomaterials, and rare-earth nanomaterials, among others (Figure 1).

### 2.1. Metal Nanomaterials

Typical metallic nanomaterials include gold nanoparticles (AuNPs) [41], silver nanoparticles (AgNPs) [42], and platinum nanoparticles (PtNPs) [43], among others. These nanoparticles typically range in size from 1 to 100 nm, offering a significantly increased specific surface area. Their surface atoms contain unsaturated bonds, providing a strong surface adsorption capacity and enabling interactions with various molecules or ions. This makes them ideal carriers for drug deliver [44,45,46,47]. Additionally, their unique surface plasmon resonance (SPR) effect and magnetic properties, such as superparamagnetism, confer distinct characteristics compared to bulk materials. These include modifications in electronic structure, optical behavior, electrical properties, and magnetic modulation, making metallic nanomaterials highly valuable in applications such as biosensing, imaging, and phototherapy [48,49,50].

### 2.2. Metal Oxide Nanomaterials

Metal oxide nanomaterials, composed of metallic elements and oxygen, represent a significant class of INPs with diverse and promising applications. Examples include zinc oxide (ZnO) [51], titanium dioxide (TiO_2_) [52], and iron oxide (Fe_3_O_4_) [53]. Many metal oxides exhibit excellent magnetic properties, high specific surface area, and notable stability and biocompatibility, making them suitable for medical imaging and drug delivery to tumor tissues. For instance, Fe_3_O_4_ is one of the few INPs approved for clinical trials [54]. Additionally, metal oxides such as TiO_2_ and ZnO demonstrate exceptional photocatalytic activity when excited by UV or visible light, generating electron-hole pairs that lead to the production of ROS. These ROS can exhibit antimicrobial effects or disrupt intracellular metabolic functions, contributing to tumor cell destruction [55,56,57,58]. Furthermore, titanium dioxide has been reported to kill tumor cells by mechanically stimulating their genome, providing an additional mechanism for tumor therapy [59].

### 2.3. Quantum Dots

Quantum dots are semiconductor nanomaterials with unique optical and electronic properties, typically composed of group II-VI elements such as cadmium sulfide (CdS) [60], cadmium selenide (CdSe) [61], and zinc sulfide (ZnS) [62]. Due to their quantum confinement effect, quantum dots exhibit size-dependent luminescence properties that are more stable than conventional fluorescent dyes. They offer high luminescence efficiency, a broad excitation spectrum, and a strong, stable fluorescence signal, making them widely applicable in bioimaging and related fields [63,64,65]. Moreover, the surface of quantum dots can be chemically modified to introduce various functional groups, such as antibodies [66], nucleic acids [67], or drugs [68], enabling targeted drug delivery. They can also be combined with magnetic nanomaterials to facilitate fluorescence-magnetic resonance multimodal imaging [69,70,71]. Some quantum dots, such as graphene quantum dots, exhibit photothermal properties under near-infrared (NIR) irradiation, enabling localized tumor ablation [72]. Red phosphorus quantum dots, upon 660 nm laser excitation, generate reactive oxygen species (ROS), exerting a photodynamic effect for tumor destruction [23]. Additionally, carbon quantum dots derived from coffee have been reported to induce ferroptosis in cancer cells and activate tumor immunity, enhancing the efficacy of immunotherapy [73]. These distinctive optical and electronic properties make quantum dots invaluable in bioimaging, drug delivery, and targeted cancer therapies.

### 2.4. Carbon-Based Nanomaterials

Carbon-based nanomaterials encompass various forms of carbon, including graphene [74], carbon nanotubes [75], and fullerenes [76]. Graphene and carbon nanotubes are among the most robust materials known, with potential applications in biomechanical scaffolds [77,78] and tissue engineering [79,80]. Their unique honeycomb structure imparts exceptional electrical and thermal conductivity, enabling them to efficiently absorb light energy and generate significant heat for photothermal tumor therapy under NIR light irradiation. Additionally, materials such as fullerenes can generate ROS, facilitating photodynamic therapy [81,82,83,84]. The honeycomb structure also provides an extremely high specific surface area, making these nanomaterials highly effective for drug loading and delivery. They can incorporate various drug molecules through mechanisms such as physical adsorption, π-π stacking, and covalent bonding modifications, allowing for controlled drug release. Their excellent biocompatibility further broadens their applicability in medical scenarios [85,86]. Furthermore, graphene oxide nanostructures have been reported to regulate tumor cell autophagy, either promoting cancer cell death or enhancing chemosensitivity, depending on the conditions [87]. Overall, carbon-based nanomaterials exhibit significant advantages in drug delivery, tissue engineering, and cancer therapy due to their unique physical, chemical, and biological properties, making them highly promising for applications in colorectal cancer treatment.

### 2.5. Magnetic Nanomaterials

Magnetic nanomaterials are nanoscale materials with magnetic properties, typically including ferrite (Fe_3_O_4_) [88], iron oxide nanoparticles (e.g., γ-Fe_2_O_3_) [89], and nanoparticles of other transition metals such as cobalt [90] and nickel [91]. These materials share similarities with previously discussed metal and metal oxide nanomaterials. Many magnetic nanomaterials exhibit superparamagnetism at room temperature, a property that allows them to become magnetized in the presence of an external magnetic field and lose magnetism once the field is removed. This characteristic prevents nanoparticle aggregation in vivo and enables rapid magnetization, making them highly valuable for magnetic resonance imaging (MRI) [92]. Magnetic nanoparticles also possess a high specific surface area, allowing for chemical modifications to incorporate functional molecules such as drugs [93], antibodies [94], nucleic acids [95], and fluorescent probes [96]. These modifications enhance the biocompatibility and multifunctionality of the nanomaterials while reducing toxicity. Additionally, magnetic nanomaterials can convert magnetic energy into thermal energy under an alternating magnetic field, enabling their use in magnetic hyperthermia for tumor treatment [97,98,99]. These unique properties give magnetic nanoparticles significant advantages in applications such as multimodal imaging, drug delivery, and targeted therapy for colorectal cancer. With continuous advancements in nanotechnology and biomedical research, the medical potential of magnetic nanomaterials is expected to expand even further.

### 2.6. Rare-Earth Nanomaterials

Rare-earth nanomaterials are nanomaterials composed primarily of rare-earth elements, including rare-earth oxides (e.g., cerium oxide [CeO_2_] [100], neodymium oxide [Nd_2_O_3_] [101]), rare-earth-doped materials (e.g., europium-doped Y_2_O_3_ [102]). These materials typically exhibit excellent stability and biocompatibility, which can be further enhanced through surface modifications or functionalization. This allows rare-earth nanomaterials to serve as multifunctional carriers for drug transport, enabling photo-responsive modulation of drug release to enhance targeted delivery and therapeutic efficacy [103,104]. Some rare-earth nanomaterials also exhibit superior magnetic resonance imaging (MRI) capabilities, particularly when combined with magnetic nanomaterials [105,106,107]. Their unique electronic structure and rich energy levels endow them with distinctive optical properties, allowing them to absorb NIR light and convert it into visible light, making them highly valuable for bioimaging and phototherapy applications [108,109]. Additionally, rare-earth nanomaterials such as cerium oxide (CeO_2_) nanoparticles possess superoxide dismutase (SOD) and catalase (CAT)-like activities, which enable them to effectively scavenge excessive free radicals in the body. This property offers significant potential for tumor therapy [110,111,112].

Overall, INPs, with their unique properties, have demonstrated significant potential in the field of medicine, particularly in tumor diagnosis and treatment. Table 2 lists the physical properties of common INPs and the application scenarios in colorectal cancer diagnosis and treatment. Fully exploring their performance advantages and advancing their clinical applications are crucial steps toward achieving personalized medicine and precision therapies. As colorectal cancer ranks as the second most prevalent solid tumor globally, improving early diagnosis and treatment is essential for enhancing patients’ quality of life. The following sections will detail recent progress in the use of INPs for the diagnosis and treatment of colorectal cancer.

## 3. Inorganic Nanomaterials in Diagnosis and Therapy of Colorectal Cancer

### 3.1. Diagnostics

#### 3.1.1. CT Imaging

**Figure 2:**
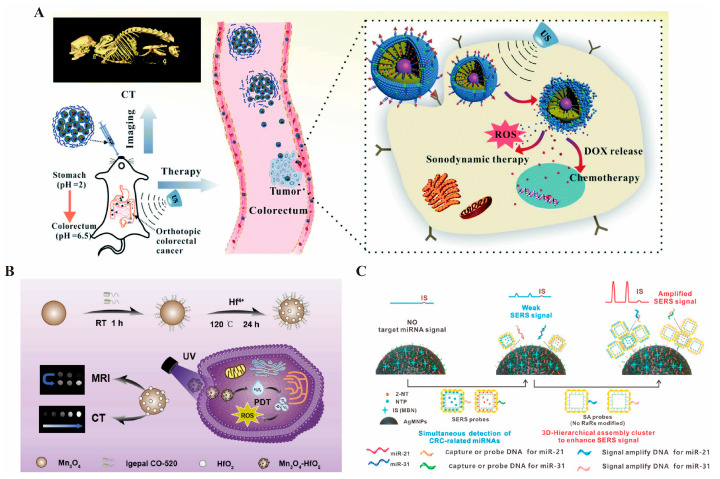
(**A**). Schematic representation of the effects of GMCDS-FA@CMC CT imaging and synergistic treatment [132] © The Royal Society of Chemistry 2021. (**B**). Schematic representation of the effects of Mn_3_O_4_-HfO_2_ preparation, MRI/CT dual imaging, and treatment [136] © 2024 Elsevier B.V. (reprinted from Ref. [136], copyright (2024), with permission from Elsevier). (**C**). Schematic of IS-AgMNPs excellent Raman imaging to capture upregulated miRNAs in colorectal cancer [213] © 2023 Elsevier B.V. (reprinted from Ref. [213], copyright (2023), with permission from Elsevier).

Diagnostic CT imaging is pivotal for staging and grading colorectal cancer. INPs, with their excellent X-ray attenuation properties, show significant potential as contrast agents for specific imaging. Metallic nanomaterials, including gold nanoparticles [196], bismuth-based nanomaterials [23], and other metallic compounds, are widely utilized in CT imaging of colorectal cancer. These materials offer superior X-ray attenuation compared to iodinated CT contrast agents, providing sharper imaging, particularly in soft tissues, organs, and tumor tissues. This improved contrast enhances the sensitivity of CT imaging [197]. Zhang et al. [132] designed and synthesized an enteric nanoprobe incorporating gold nanoparticles, which demonstrated exceptional CT imaging performance (Figure 2A). Similarly, bismuth-based nanoparticles exhibited remarkable advantages, including enhanced contrast and prolonged imaging time. Bismuth, commonly used as a mucosal protectant in gastrointestinal diagnostics, has been proven safe through extensive clinical use, making it a promising material for diagnostic and therapeutic integration [23]. Shakeri et al. [198] developed a combination of elemental Bi and iodine to construct BiOI nanoparticles, a contrast agent that compensates for the X-ray attenuation effect of elemental iodine alone, yielding multiples of the effect of CT imaging to obtain a clearer view of the field of view and the extent of the tumor.

In addition to metal nanoparticles, certain rare-earth nanomaterials, such as lanthanum, cerium, and gadolinium, exhibit significant X-ray attenuation effects. Compared to iodine-based contrast agents, these elements demonstrate better compatibility with X-rays, resulting in stronger imaging contrast, making them promising candidates for CT contrast agents [199,200]. Shi et al. [131] developed multifunctional transferrin-encapsulated GdF_3_ nanoparticles (64Cu-GdF_3_@Tf-Cy7 NPs), which not only enabled specific CT and MRI imaging of colorectal cancer with high transferrin expression but also allowed precise characterization of sentinel lymph nodes, demonstrating remarkable potential. However, the biosafety of INPs must be prioritized in clinical trials. In this regard, gold and bismuth nanomaterials, with their multifunctional therapeutic properties, offer promising solutions for future applications.

#### 3.1.2. MRI

Metal elements currently approved for intravenous use in clinical practice as MRI contrast agents fall into three categories: gadolinium-based, manganese-based, and iron-based agents [201]. Among these, gadolinium-based contrast agents are the most widely used. Gadolinium exhibits strong paramagnetism and alters the magnetic field environment of surrounding tissues in vivo [202]. This effect shortens the T1 and T2 relaxation times of local protons, increasing the T1-weighted image (T1WI) signal intensity while decreasing the T2-weighted image (T2WI) signal intensity. Similarly, iron oxide nanoparticles demonstrate enhanced longitudinal (T1) and transverse (T2) relaxation, enabling superior imaging in both T1 and T2 contrasts, particularly in low magnetic field environments [203]. At 1.5T, the longitudinal relaxivity (r1) of iron oxide in biological media is measured at 19.0 mM^−1^ s^−1^, while its transverse relaxivity (r2) reaches 64.9 mM^−1^ s^−1^, significantly higher than that of commonly used gadolinium-based contrast agents [204]. Due to their improved biocompatibility and biosafety compared to gadolinium- and manganese-based agents, iron oxide nanoparticles are increasingly regarded as promising MRI contrast agents. Additionally, their potential application in anemia treatment further enhances their clinical appeal [205].

Manganese-based nanomaterials also exhibit strong paramagnetic properties, effectively altering the longitudinal relaxation of water molecules. They are metabolized safely in vivo, offering excellent biocompatibility [136,206] (Figure 2B). MRI studies in various cell lines have demonstrated that the use of manganese contrast agents significantly increases signal intensity in T1WI. Specifically, the mean T1 values of SW620 and LoVo cancer cells were shortened to 289.33 ± 0.57 ms and 268.45 ± 6.87 ms, respectively, compared to normal cells [206]. This notable reduction in T1 imaging time makes manganese-based agents particularly effective in cancer imaging. Additionally, a comparative study by Wen et al. [207] evaluated the contrast-enhancing properties of manganese- and gadolinium-based MRI agents, revealing that manganese-enhanced MRI provides superior contrast and a larger enhancement area. In tumors of 5, 10, and 15 mm, manganese significantly shortened the T1 relaxation times by 513.72, 205.9, and 275.09 ms, respectively, compared to gadolinium. Moreover, bismuth–gadolinium was shown to be particularly advantageous for early detection of small lesions [208].

#### 3.1.3. Raman Endoscopic Imaging

Raman imaging is based on the Raman scattering effect, where laser light irradiates tissues containing nanomaterials, causing a change in the frequency of the scattered light. This produces specific Raman scattering spectra with unique spectral characteristics for different nanomaterials that serve as “fingerprints” for identification. By collecting and analyzing Raman spectral data, highly chemically specific images of the intestinal interior can be constructed, enabling precise differentiation between tumor and normal tissues, even in areas with subtle compositional differences [209]. Compared with traditional white-light endoscopy, Raman scattering imaging can detect smaller precancerous lesions, minute malignant tumors, and hidden abnormalities. Leveraging the growth characteristics of gastrointestinal tumors, Raman endoscopy can locate malignant lesions at earlier stages, providing an opportunity for timely intervention and potential cure. Surface-Enhanced Resonance Raman Scattering Nanoparticles (SERRS-NPs) are designed to enhance Raman sensitivity and improve imaging quality [210]. AuNPs are widely used in fluorescence imaging and surface-enhanced Raman scattering (SERS) due to their superior optical properties. Literature reports have shown that one-step synthesis of Au-Ag bimetallic nanostructures with sharp buds can achieve high yields, increasing surrogate concentrations to less than 1 pM and significantly enhancing imaging effects [211]. Additionally, loading anti-CEA antibodies onto SERS-modified magnetic nanoparticles allows specific binding to cells with high CEA expression, enabling precise imaging of these cells during Raman endoscopy. This approach serves as a diagnostic tool for CEA-expressing tumors, micro metastases, and tumor cycle studies [212]. Beyond conventional colorectal cancer markers, Raman imaging is also well suited for analyzing promising miRNA biomarkers in serum, which is critical for early diagnosis and prognosis of colorectal cancer (CRC). Wu et al. [213] (Figure 2C) developed a novel SERS strategy based on three-dimensional hierarchically assembled clusters. These clusters include SERS probes (AuNC@Au NPs), magnetic capture units (AgMNP), and signal amplification probes (SA probes), enabling dual enrichment and enhancement. This strategy provides ultrasensitive and quantitative analysis of upregulated miRNAs in CRC.

#### 3.1.4. Photoacoustic Imaging

Photoacoustic imaging integrates the advantages of optical and ultrasound imaging. When a short-pulse laser irradiates a tissue region, the absorbed light energy causes thermal expansion in different tissues, generating ultrasonic signals. These signals are detected by an ultrasound transducer, converted into electrical signals, and used to reconstruct an image of the tissue’s interior. This method combines the high contrast of optical imaging with the deep penetration capability of ultrasound imaging, enabling effective visualization of deep-seated tumors. Moreover, it circumvents the severe signal attenuation encountered in deep tissues with pure optical imaging [214,215].

**Figure 3:**
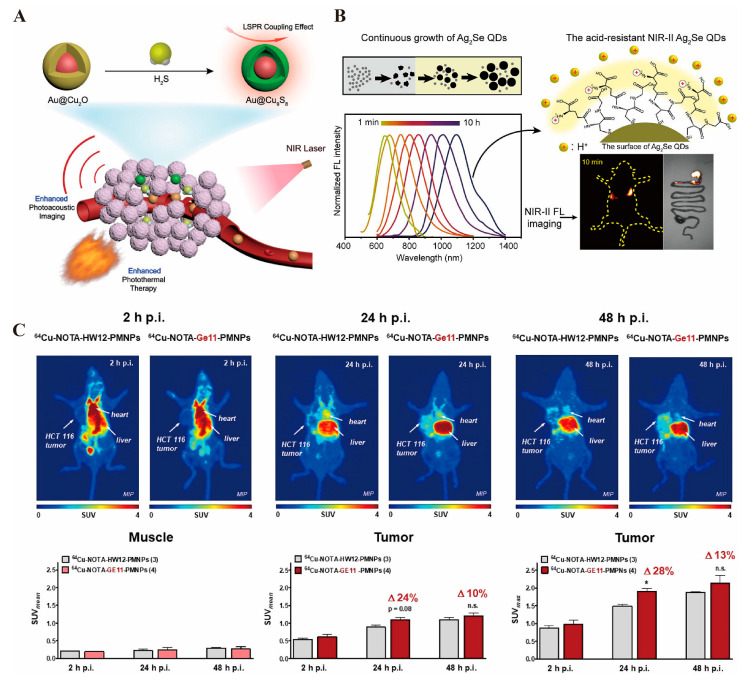
(**A**). Schematic preparation of Au@Cu_9_S_8_, which can realize enhanced photoacoustic imaging and photothermal therapy under NIR light activation [217] © 2019 WILEY-VCH Verlag GmbH & Co. KGaA, Weinheim. (**B**). Schematic of Ag_2_Se QDs, which can realize precise fluorescence imaging under NIRII photoactivation [224] © 2023 American Chemical Society. (**C**). ^64^Cu-labeled GE11-modified polymeric micellar nanoparticles enable targeted uptake by colorectal cancer for high-resolution PET imaging results [239] © 2020 American Chemical Society. * *p* < 0.05, n.s. represents no statistically significant difference in *p* values.

Gold nanorods possess exceptional photothermal conversion capabilities, efficiently converting absorbed light energy into heat, which induces the thermal expansion of the surrounding medium and generates ultrasonic signals for photoacoustic imaging. These nanorods also exhibit excellent stability and biocompatibility, maintaining their structural integrity within the biological environment, thereby minimizing adverse immune responses or other side effects [216]. To optimize their application in vivo for gastrointestinal tumor imaging, Tao et al. developed acousto-optic imaging particles by coupling gold nanoparticles with cuprous oxide. This design enhances photothermal conversion efficiency and delivers consistent imaging results even at low injection volumes [217] (Figure 3A). Copper nanoparticles, similarly, demonstrate excellent NIR light absorption properties, producing significant localized photothermal effects under NIR laser irradiation. These effects not only generate photoacoustic signals but also improve imaging contrast by altering the local temperature of gastrointestinal tumor tissues, facilitating clearer differentiation between tumor tissues and surrounding normal tissues [218].

#### 3.1.5. Fluorescence Imaging

Fluorescent nanomaterials, including quantum dots and fluorescent nanoparticles, are extensively utilized in the fluorescence imaging of colorectal cancer due to their high fluorescence quantum yield and stable optical properties [149]. Through surface modifications, these nanomaterials can specifically bind to tumor cells, either on their surface or within, enabling precise tumor labeling and imaging [219].

The unique structure of quantum dots confines electron movement in all three dimensions, resulting in the quantum confinement effect [220]. Quantum dots feature a broad excitation spectrum and a narrow, symmetric emission spectrum, enabling excitation by a single light source to produce emissions at multiple wavelengths [221]. This sharp, high-resolution emission spectrum facilitates easy detection and differentiation in complex biological samples. Additionally, quantum dots exhibit a high fluorescence quantum yield and intense luminescence, generating strong fluorescence signals even at low concentrations, thereby enhancing imaging sensitivity [222]. Their excellent photostability surpasses that of traditional organic fluorescent dyes, as they are resistant to photobleaching under prolonged illumination. This stability ensures consistent and accurate imaging over time [223]. Yang et al. [224] developed an Ag_2_Se quantum dot (Figure 3B) with remarkable stability and fluorescence continuity in gastric acid, enabling long-term monitoring of intestinal peristalsis. Compared to conventional barium meals, these quantum dots provided clearer intestinal imaging with superior biocompatibility.

Fluorescent nanoparticles encompass a wide range of types, including carbon fluorescent nanoparticles and polymer fluorescent nanoparticles [225,226]. In intestinal endoscopy, the diagnosis of malignant lesions using white-light endoscopy depends heavily on the operator’s skill and experience, and early-stage malignant lesions—such as those seen in Lynch syndrome—are often missed. This issue is exacerbated when lesions are hidden behind mucosal folds or are undetectable by standard endoscopy, making early detection challenging. While local staining could improve diagnostic accuracy, it is cumbersome and time-consuming, highlighting the need for better solutions [227]. Silica fluorescent nanoparticles offer several advantages, including good biocompatibility, ease of surface modification, and high stability [228]. Their surface can be easily functionalized with various targeting molecules, such as antibodies and peptides, enabling specific recognition and binding to intestinal tumor cells [229]. Moreover, the fluorescent dyes encapsulated within these nanoparticles are better protected, reducing the impact of external environmental factors on their fluorescence performance [230]. Rogalla et al. synthesized fluorescent silica nanoparticles that were validated in animal models, demonstrating their ability to highlight the location of adenomas in colorectal adenomas, as well as to show proliferative polyps unrelated to adenocarcinomas, all while exhibiting low biotoxicity [231].

#### 3.1.6. PET Imaging

Positron emission tomography (PET) relies on the injection of a positron-radionuclide-labeled tracer into the body. In tumor tissue and other metabolically active regions, positrons interact with electrons, leading to annihilation radiation that produces a pair of gamma photons. These photons are detected by the scanner and used to create an image reflecting the tumor’s metabolic activity, proliferation, and other functional characteristics [232]. CT uses X-ray imaging to clearly depict the anatomical structure of the gastrointestinal tract. Nanomaterials, such as gold nanoparticles [233], enhance CT imaging by altering the local tissue’s attenuation properties, making tumor tissues more visible. Additionally, they can carry radionuclides for PET imaging, reflecting tumor metabolism and linking the precise anatomical data from CT with the functional metabolic information from PET [234]. Magnetic resonance imaging (MRI) offers detailed anatomical images as well. When combined with nanomaterials (e.g., magnetic iron oxide nanoparticles [235], quantum dots [236]), MRI benefits from enhanced anatomical imaging through the nanomaterials’ MRI-detectable properties, while the radionuclide labeling allows for PET functionality. This combination enables the integration of both metabolic and anatomical data for more accurate diagnosis and assessment [237]. A novel nanomaterial designed with glucose-modified dendritic macromolecule-embedded gold nanoparticles (Au DENPs) labeled with the radionuclide 68Ga and doped with cytosine–guanine (CpG) oligonucleotides has been developed for dual-mode imaging (PET/CT) and tumor immunotherapy. Compared to other materials, Au DENP shows greater sensitivity in diagnostics and an inhibitory effect on tumors [238]. Paiva et al. [239] designed a nano-colloid containing a GE11 peptide labeled with the radionuclide 64Cu for precise, targeted imaging of colorectal cancer, demonstrating rapid renal metabolism (Figure 3C). INPs offer diverse diagnostic applications in colorectal cancer, and the future of tumor diagnosis lies in multimodal imaging that incorporates various types of INPs.

### 3.2. Drug Delivery

In cancer therapy, various organic materials, such as liposomes [240], micelles [241], and other nanodrug delivery systems, have been developed with increasing sophistication. Some of these have already received FDA approval for clinical use, largely due to their significant applications during the COVID-19 pandemic. For instance, the mRNA vaccine, which encapsulates mRNA in liposomes, was awarded the Nobel Prize in Physiology or Medicine in 2023 [242]. However, conventional organic nanomaterials still face challenges, such as low drug-loading capacity, drug leakage, and an inability to efficiently control drug release [243]. Furthermore, these treatments are heavily reliant on the drugs they deliver, which are susceptible to drug resistance. This has led to the development of numerous INPs designed to achieve more precisely targeted drug delivery and controlled release.

Due to their inherent properties, many INPs can efficiently load therapeutic drugs without requiring additional modifying groups. Iranpour et al. [244] successfully loaded doxorubicin (DOX) and graphene quantum dots (GQDs) with a loading capacity of up to 90% using the ZIF-8 platform. To enhance the platform’s safety and targeting ability, they modified the surface with polyethylene glycol (PEG) and the EpCAM aptamer, an epithelial cell adhesion molecule. This modification enabled a synergistic treatment strategy for colorectal cancer, combining radiotherapy, chemotherapy, and targeted therapy. Mesoporous silica nanoparticles (MSNs) exhibit excellent biocompatibility, high porosity, and ease of functionalization. Their large surface area and pore volume make them highly effective for drug adsorption and loading [245]. A recent study reported that MSNs achieved an impressive encapsulation efficiency of 92.13% and a drug-loading capacity of 8.42% [246]. Additionally, Song et al. designed bismuthene nanomaterials with an exceptional DOX loading capacity of approximately 250% and encapsulation efficiency of 59.14% [23]. In comparison, liposomes demonstrated a lower encapsulation efficiency of 88.56% and a drug-loading capacity of only 3.03% [247].

Despite these advances, some INPs still face challenges in terms of loading capacity. For instance, materials like graphene have superior loading potential, while other inorganic materials exhibit less efficient drug loading [82]. Moreover, the nonspecific toxicity of certain inorganic materials, driven by the pure EPR effect, can increase toxicity [248]. However, surface modification with various aptamers and moieties can enhance both loading and targeting abilities, reducing toxicity while improving therapeutic outcomes. Lee et al. [249] designed AuNPs with a diameter of 13 nm and wrapped them with GC-rich oligonucleotides (ONTs). This modification provided a number of binding sites for Adriamycin (DOX). The resulting drug delivery platform showed potential for colorectal cancer therapy. Go et al. [250] combined gold nanoparticles with a cellular prion protein aptamer (PrPC-APT) and loaded DOX, which inhibited mitochondrial function in colorectal cancer cells, decreasing peroxisome proliferator-activated receptor gamma coactivator 1-alpha expression and oxygen consumption rate, thus suppressing cancer cell proliferation. Modified INPs like these dominate current drug delivery platforms, playing a central role in the development of effective cancer therapies.

Accurately targeting tumor sites to reduce off-target toxicity remains the primary challenge for the development of INPs. To achieve this, surface modifications using various aptamers—such as folic acid, hyaluronic acid, peptides, and nucleic acid aptamers—are commonly employed. Special membranes, including those derived from cancer cells, are also being developed for enhanced targeting [251,252,253,254]. These aptamers exploit the principle of antigen–antibody pairing, promoting high enrichment at tumor sites while minimizing aggregation in normal tissues, resulting in effective localized tumor-killing effects. One prominent targeting strategy is the modification of INPs with folic acid (FA), as tumor cells often overexpress folate receptors. This modification significantly enhances tumor targeting. Jaiswal et al. modified bovine serum albumin with folic acid on gold nanorods to deliver therapeutic RNaseA for colorectal cancer treatment, achieving excellent therapeutic outcomes [255]. Another common target in colorectal cancer cells is integrin αvβ, which contains the RGD-binding domain. Pan et al. [256] targeted colorectal cancer by modifying MSNs with the RGD-binding peptide, improving the anticancer effects of 5-fluorouracil (5-FU). Additionally, the hyaluronan receptor CD44 and the nucleolin receptor are abundantly expressed in colorectal cancer and other tumor cells. Therefore, hyaluronan and nucleolin aptamer AS14AA are frequently used to enhance the targeting properties of INPs. For example, Hu et al. [257] used calcium carbonate nanoparticles to deliver curcumin (Cur) and the protein deacetylase inhibitor QTX125. They modified the surface of these nanoparticles with hyaluronic acid, improving their targeting ability and anticancer effects in colorectal cancer cells. Similarly, Hassibian et al. [258] constructed Apt-CCM-HG@MTX, a system where hollow gold nanoparticles carrying methotrexate (MTX) were surface-modified with the nucleic acid aptamer AS1411. This modification enabled enhanced targeting of cancer cells and synergistically boosted the therapeutic effects of chemotherapy and photothermal therapy under NIR light irradiation. These strategies demonstrate that surface modification with targeting ligands, such as folic acid, RGD peptides, hyaluronic acid, and nucleolin aptamers, plays a crucial role in enhancing the precision and effectiveness of INPs in cancer therapy. By ensuring selective accumulation at the tumor site, these modifications help to minimize off-target toxicity and improve therapeutic outcomes.

In addition to surface modification with aptamers, another highly effective and promising strategy for improving the targeting and delivery of INPs is direct encapsulation within the cell membranes of target cells. This method has garnered attention for its ability to enhance biocompatibility, target specificity, and drug delivery efficiency. For instance, Liu et al. [259] designed a hybrid MnO_2_/PDA “nano-bomb” encapsulated in macrophage membranes. This system not only effectively targeted colorectal cancer but also exhibited chemo-dynamic, photodynamic, and photothermal therapeutic properties, along with excellent biodegradability. While theoretically, the cell membranes of different cell types can be used for encapsulating INPs, there are some challenges to consider. Specifically, it is crucial to address potential tumor-promoting factors present on the surface of malignant cells, which may affect the effectiveness of the delivery system. As our understanding of various molecular proteins and cell membrane characteristics increases, the application of membrane-encapsulated INPs will likely play an increasingly significant role in drug delivery and tumor targeting. For specific applications, such as oral drug delivery for gastrointestinal cancers, traditional surface modifications or membrane coatings may not suffice due to the harsh environment of the stomach, where gastric acid can degrade drugs. In these cases, additional strategies are required to protect the drugs from acid corrosion and ensure they reach the intestines effectively. One solution is the use of acid-resistant coatings, such as chitosan, which can prevent the degradation of drugs in the stomach and allow for targeted delivery in the intestinal tract [260]. Another promising approach involves pH-responsive designs that release the drug in the tumor environment, which is often more acidic than normal tissues. Certain INPs, such as silicon dioxide, are particularly well suited for this purpose due to their excellent chemical stability, allowing them to protect drugs from stomach acid while delivering them effectively to tumor sites [261]. These advanced strategies, combining membrane encapsulation with pH-sensitive coatings or other protective materials, are crucial for enhancing the effectiveness of drug delivery systems, particularly for treatments targeting the gastrointestinal tract.

### 3.3. Therapy

#### 3.3.1. Photothermal Therapy

Photothermal therapy (PTT) is an emerging technique for tumor treatment that shows great potential in colorectal cancer therapy. It selectively kills tumor cells by irradiating the tumor site with NIR light of specific wavelengths, activating targeted photothermal conversion materials (e.g., gold nanoparticles [262], graphene oxides [263], carbon nanotubes [264]), which convert light energy into heat, inducing localized high-temperature damage to tumor cells. This treatment offers several advantages, including non-invasiveness, high efficiency, and precision [265]. The effectiveness of photothermal therapy heavily depends on the use of photosensitizers, which must possess key properties such as strong light absorption, high photothermal conversion efficiency, and biocompatibility [266]. INPs, particularly metallic nanomaterials, excel in photothermal therapy due to their exceptional optical properties.

**Figure 4:**
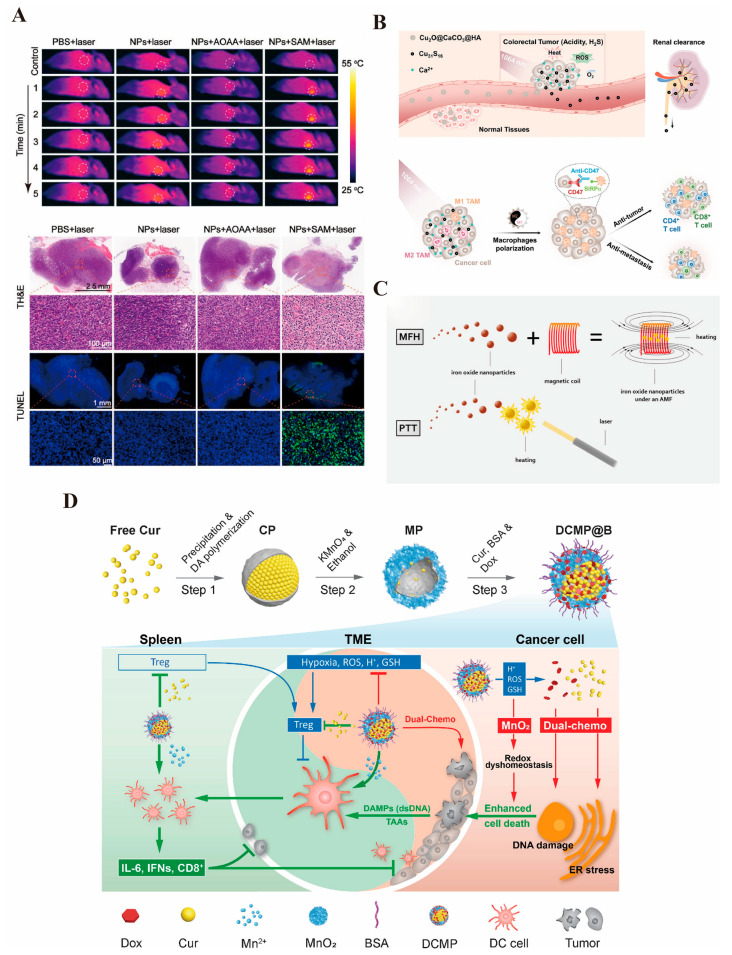
(**A**). The photothermal effect of Bi:Cu_2_O@Ha can reach close to 50 °C and has an excellent killing effect on colorectal cancer [271] © 2022 The Authors. Journal of Biomedical Materials Research Part A published by Wiley Periodicals LLC (New York, NY, USA). (**B**). Cu_2_O@CaCO_3_, schematic representation of the effect of photothermal/photodynamic/immunotherapeutic treatments, and can be safely metabolized by the kidneys with good biological safety [273] © 2020 Wiley-VCH GmbH (Wernheim, Germany). (**C**). Schematic illustration of the superior effect of magnetic and photothermal iron oxide nanoparticles [141]. (**D**). Schematic representation of the preparation of the MnO_2_ drug-carrying platform and the immune-activating effect produced by delivering DOX and curcumin to the colorectal cancer site [144] © 2022 Elsevier Inc. (Amsterdam, The Netherlands) (reprinted from Ref. [144], copyright (2022), with permission from Elsevier).

In colorectal cancer, photothermal therapy not only directly kills cancer cells but also enhances the therapeutic effect by combining strategies such as targeted drug release or immune modulation. Additionally, with the continuous optimization of photothermal conversion materials in recent years, photothermal therapy has gradually achieved precise regulation of the tumor microenvironment and significantly reduced damage to surrounding normal tissues [267]. As mentioned earlier, gold nanomaterials exhibit exceptional optical properties due to the unique surface plasmon resonance (SPR) effect on their surfaces, and their photothermal properties are particularly remarkable. Many studies have reported the excellent photothermal effects of gold nanoparticles in the targeted therapy of colorectal cancer [268,269,270]. Single-walled carbon nanotubes (SWNTs), which are cylindrical graphite helical molecules, exhibit excellent photothermal properties under NIR light irradiation. Chen et al. [271] constructed ADP@SWNT nanomaterials by coupling third-generation alkyne-focused poly(L-lysine) dendrimer (PLLD-G3) with SWNTs via click chemistry, demonstrating remarkable photothermal performance in the treatment of colorectal cancer. Cheng et al. [272] (Figure 4A) designed Bi:Cu_2_O@HA nanoparticles with a hyaluronic acid coating to deliver H_2_S gas targeted delivery to reach the colorectal cancer site. The doped Bi serves as a reagent for CT imaging and enhances the photothermal properties of H_2_S-triggered Cu_2_O, producing excellent results, which are summarized in the following table. The development of photothermal therapy has greatly facilitated the application of INPs in colorectal cancer, addressing challenges posed by deep and drug-resistant tumors. Furthermore, the combination of photothermal therapy with immunotherapy and chemotherapy further enhances the anti-tumor effect. It has been shown that MoSe_2_, in addition to its powerful photothermal properties, can deplete glutathione, promote the release of tumor-associated antigens, and induce immunogenic cell death (ICD) to enhance immunotherapy, thereby significantly inhibiting colorectal cancer progression [273].

#### 3.3.2. Photodynamic Therapy

Photodynamic therapy (PDT) is a tumor treatment technique that involves the interaction of a photosensitizer, a specific wavelength light source, and oxygen, demonstrating significant potential in the treatment of colorectal cancer [274]. PDT induces oxidative damage and apoptosis in tumor cells by selectively accumulating photosensitizers in tumor tissues and activating them under light irradiation at specific wavelengths to generate singlet oxygen and other ROS [275]. Additionally, PDT destroys tumor blood vessels and activates anti-tumor immune responses, further inhibiting tumor growth and metastasis [276].

In the treatment of colorectal cancer, photodynamic therapy (PDT) has garnered significant attention due to its advantages of minimal trauma, low toxicity, and reduced damage to normal tissues. Recent advancements in photosensitizers and light source technology have enhanced PDT’s targeting capabilities and therapeutic efficiency in the precision treatment of colorectal cancer [277]. Many other solid tumors are located deeper within the body, limiting the application of photothermal and photodynamic therapies since external light sources cannot directly penetrate the skin and internal organs to reach the target site [278]. However, because the intestinal lumen is connected to the outside world, light sources can be directly irradiated to the targeted tumor site, facilitating the application of PDT in colorectal cancer. Numerous studies have explored the use of INPs, such as metal nanoparticles and oxides, in colorectal cancer treatment [279,280,281,282] (Figure 4B). Nevertheless, PDT requires an aerobic microenvironment and is less effective in an anaerobic one. As a result, more studies are investigating how INPs can be utilized to generate therapeutic benefits in the anoxic environment of colorectal cancer. Cui et al. [136] designed a manganese–hafnium nanocomposite (Mn_3_O_4_-HfO_2_ NCs) that not only enables multimodal imaging for MRI/CT but also reacts with hydrogen peroxide in tumor cells to generate a large amount of ROS, thereby killing the tumors while exhibiting excellent biocompatibility. Wang et al. [283] developed a PEG-modified V-MoS_2_@PEG nano-enzyme that, under NIR laser irradiation, releases drugs. The molybdenum ions (Mo^4+^) react with hydrogen peroxide to generate hydroxyl radicals, while vanadium ions deplete glutathione, preventing the depletion of hydroxide ions and producing significant heat, which leads to a synergistic therapeutic effect and improves the therapeutic efficiency in colorectal cancer. Additionally, PDT can be combined with conventional treatments such as surgery, chemotherapy, or immunotherapy to further enhance the overall therapeutic effect. It has been reported that Fe_3_O_4_ nanoparticles generate excellent photothermal effects, catalyze the generation of ROS by depleting glutathione, and enhance PTT-induced immunogenic cell death through Fenton reactions, which increases CD8+ T-cell infiltration into tumors, achieving a synergistic therapeutic effect [284].

Phototherapy, as a non-invasive treatment method, has shown considerable potential in both research and clinical applications for colorectal cancer. The exceptional properties of INPs have paved the way for the application of phototherapy, and the development of multimodal and combined therapies has become a key component of personalized treatment for colorectal cancer.

#### 3.3.3. Magnetic Hyperthermia Therapy

In recent years, with the growth of phototherapy, researchers have begun exploring various potential therapeutic modalities. Magnetic hyperthermia therapy (MHT) is gaining attention within the scientific community [285]. By applying an alternating magnetic field, magnetic nanoparticles (MNPs) generate local heat, converting magnetic energy into thermal energy. This raises the temperature of tumor tissue, inducing apoptosis or necrosis in tumor cells. Additionally, magnetic nanoparticles can be used for in vitro MRI imaging, enabling the integration of diagnosis and treatment [286]. Iron oxide nanoparticles are particularly effective for both MRI imaging and magnetothermal therapy and have been widely applied in the treatment of colorectal cancer [141] (Figure 4C). Fernandes et al. [287] utilized iron oxide nano-cubes as MHT media, coated with thermo-responsive substances (TR-tubes) and loaded with the chemotherapeutic agent DOXO. This approach achieved magnetic field-controlled thermotherapy and chemotherapy, effectively killing colorectal cancer stem cells. Magnetothermal therapy can also activate anti-tumor immune responses by releasing tumor-associated antigens and promoting immune cell infiltration [288]. Moreover, when combined with artificial intelligence and big data analysis, the parameters of MHT—such as magnetic field strength, frequency, and nanoparticle concentration—can be personalized to suit different tumor types and optimize treatment according to the patient’s tolerance [289]. These multimodal diagnostics and personalized synergistic therapies offer promising prospects for the use of magnetic nanomaterials.

#### 3.3.4. Immunotherapy

The drug-resistant and immunosuppressive microenvironment of colorectal cancer has long been a significant barrier to effective treatment [290]. In some cases, such as with tumors causing intestinal obstruction, direct delivery through colonoscopy may not be feasible, and the dense tumor stroma prevents drugs from reaching the deeper layers of tumor cells [291]. Therefore, improving the immunosuppressive or dense microenvironment is crucial to overcoming this challenge. As previously discussed, INPs, which serve as excellent drug carriers, can exert anticancer effects by delivering immune activators to the tumor site, thus stimulating anti-tumor immune responses. Liu et al. [144] developed an innovative MnO_2_ nanoplatform that delivers DOX and curcumin (CUR), activating the immune system to target and kill colorectal cancer cells (Figure 4D). Nanoparticle-based immune adjuvants can enhance antigen presentation and stimulate T-cell activation by delivering tumor-associated antigens (TAAs). Additionally, inorganic materials such as copper oxide can act as immunomodulators to activate immune-related pathways within tumor cells, modulate the immune microenvironment, and improve the efficacy of tumor immunotherapy. Lin et al. [292] synthesized Cu_2_O@Au nanomaterials to induce ferroptosis in colorectal cancer cells, thereby promoting dendritic cell (DC) maturation and T-cell infiltration, which enhanced the anti-tumor effects of PD-L1.

## 4. Biocompatibility and Toxicity of Inorganic Nanomaterials

The extensive application of INPs in the treatment of colorectal cancer has raised significant concerns regarding their biosafety and potential toxicity, both of which are crucial factors influencing therapeutic efficacy. The accumulation of metallic nanomaterials in the body may lead to interactions with intracellular components, potentially disrupting cellular functions and inducing metal toxicity [293]. This toxicity is primarily attributed to their small particle sizes and large specific surface areas, which increase their reactivity and potential for biological interactions [294]. Therefore, INPs used in colorectal cancer treatment must actively adapt to various physiological conditions in the human body while maintaining safety as they pass through different biological barriers [295]. Since human blood is negatively charged, nanomaterials must also carry a negative zeta potential; positive zeta potential nanoparticles can cause severe adverse reactions. To address this, surface modifications are often introduced to ensure a final negative charge, thereby enhancing stability in circulation [296]. Additionally, during blood circulation, INPs must evade recognition and clearance by the immune system to maximize drug accumulation at the tumor site, ultimately improving therapeutic efficacy [297]. Moreover, the tumor microenvironment is typically slightly acidic (pH~6.5) compared to normal tissues (pH~7.4). This necessitates that INPs release drugs or activate their therapeutic functions in response to this acidic environment [298]. Furthermore, tumor tissues are often rich in reducing agents such as glutathione (GSH), which can be leveraged to enable controlled drug release and targeted therapy [299].

To adapt to these physiological conditions and enhance the biocompatibility of INPs while minimizing adverse effects, various chemical strategies have been developed. One widely employed approach is surface modification, where nanomaterials are functionalized with biocompatible coatings such as polyethylene glycol (PEG) [300], dextran [301], or phospholipids [302]. These coatings improve colloidal stability, reduce nonspecific protein adsorption, and minimize immune recognition, thereby decreasing cytotoxicity. Furthermore, the development of responsive nanomaterials, which can release drugs in response to factors like pH, temperature, magnetic fields, or light, holds promise for reducing toxicity to normal tissues by enabling targeted drug release at colorectal cancer sites. Another effective strategy is core–shell engineering, in which an inert or biocompatible material is coated onto the nanoparticle surface to serve as a protective barrier [303]. Coating quantum dots with a silica or ZnS shell effectively prevents the release of toxic metal ions, thereby significantly reducing cytotoxicity [304,305]. Additionally, doping and elemental substitution have been explored to modulate the physicochemical properties of nanomaterials and reduce toxicity [306]. Furthermore, the development of biodegradable nanomaterials offers a promising approach to minimizing toxicity concerns [307]. Biodegradable silica and calcium phosphate nanoparticles gradually degrade into non-toxic byproducts under physiological conditions, ensuring safe clearance from the body and reducing the risk of long-term accumulation [308,309]. In addition, some inert metal materials, such as bismuth nanoparticles [310], which are easily metabolized by the body, have been widely used and validated in clinical applications. For example, bismuth-based agents are commonly used to treat gastric ulcers [311]. These nanomaterials can be excreted from the body through the kidneys and serve as excellent drug carriers for clinical development and application. Collectively, these chemical strategies play a vital role in improving the biosafety and therapeutic potential of INPs, making them more suitable for biomedical applications.

## 5. Discussion and Conclusions

Theranostics, a concept that integrates both diagnostic and therapeutic functions into a single nanomaterial or system, holds significant promise for colorectal cancer diagnosis and treatment. By combining diagnostic imaging and therapeutic action, theranostics enables personalized, real-time monitoring of both treatment efficacy and drug delivery, ensuring that therapy is tailored to the individual patient’s needs. The ability to track and assess therapeutic progress non-invasively with theranostic nanoparticles presents a major advancement in colorectal cancer care. It allows clinicians to adjust treatment protocols quickly based on real-time imaging data, leading to more accurate and efficient treatment decisions. As a result, theranostics offers the potential to significantly improve clinical outcomes in colorectal cancer patients by enabling personalized, effective therapies.

INPs have achieved remarkable results in colorectal cancer research due to their unique physicochemical properties, such as high stability, easy functionalization, and excellent photothermal and photosensitizing abilities [312]. To date, various INPs have been used in the theranostics of colorectal cancer. These include iron oxide nanoparticles capable of MRI diagnosis as well as photothermal or magnetic hyperthermia treatment [313], gold nanoparticles used for CT diagnosis and photothermal therapy [44], and MSN particles for MRI and PET imaging, along with controlled drug delivery [314]. The application of these INPs has advanced the integration of diagnosis and treatment in colorectal cancer and other tumors. Studies have shown that iron oxide, silica, and gold nanoparticles can serve as efficient drug delivery platforms, targeting chemotherapeutic agents like 5-fluorouracil or doxorubicin to colorectal cancer cells [135,315,316,317]. This significantly improves the cellular uptake rate and anti-tumor activity of the drugs. Apoptosis has been successfully induced in gastric and colorectal cancer cells using rare-earth-doped up-conversion nanoparticles (UCNPs) and gold nanorods, which generate thermal effects or reactive oxygen species upon laser irradiation [318]. Magnetic nanoparticles, when combined with chemotherapeutic drugs and immune adjuvants, demonstrate a synergistic effect, enhancing cancer cell killing and reducing the required chemotherapeutic drug dosage [319]. Metal oxide nanoparticles, such as Fe_3_O_4_ [320], are commonly used in MRI-guided magnetothermal therapy, while nanomaterials like bismuth and manganese are frequently used in CT-guided photothermal therapy, enabling precise localization and thermal effects [23,69].

Clinical research on INPs is progressing rapidly, with preliminary findings highlighting their potential in colorectal cancer treatment. Multimodal imaging-guided synergistic therapy represents the future direction of colorectal cancer treatment. Photodynamic therapy (PDT) for colorectal cancer showed that the photothermal effect of gold nanorods, combined with conventional chemotherapy, significantly prolonged progression-free survival [318]. In Germany, NanoTherm^®^ has been approved for an exploratory trial in gastrointestinal tumors based on its use in treating recurrent gliomas. Preliminary data suggest that magnetothermal therapy, when combined with radiotherapy and chemotherapy, significantly improves efficacy and provides some survival benefits, particularly in patients with refractory gastric cancer [321].

In addition, AI holds great potential in the application of INPs for cancer diagnosis and therapy, offering significant advancements in diagnostic accuracy, therapeutic optimization, and personalized medicine [322]. In terms of diagnosis, AI can integrate medical imaging analysis, pathological examination, and multi-omics data to accurately identify tumor characteristics, thereby enhancing the sensitivity and specificity of early cancer detection. Additionally, AI can analyze the biodistribution, metabolism, and biocompatibility of nanomaterials in vivo, facilitating the rational design of nanoplatforms with improved targeting efficiency and biosafety [323]. In the therapeutic domain, AI can leverage vast clinical datasets and in vitro experimental results to predict the pharmacokinetics and therapeutic efficacy of INPs, aiding in the development of personalized nanomedicine strategies. For instance, AI-driven machine learning models can analyze patients’ gene expression profiles to identify optimal nanocarriers and drug combinations tailored to specific tumor types, thereby enhancing treatment efficacy while minimizing adverse effects [324]. Furthermore, AI-powered intelligent nanorobots hold promise for real-time monitoring of the tumor microenvironment and dynamically adjusting drug release in response to disease progression, thereby improving the precision of cancer treatment [325]. Looking ahead, AI can facilitate the intelligent design of INPs by employing deep learning and computational simulations to optimize nanostructures for enhanced stability, targeting capability, and responsiveness to external stimuli such as light or magnetic fields. Overall, the integration of AI will drive innovation in the application of INPs for cancer diagnosis and therapy, making these approaches more precise, efficient, and personalized.

Although INPs have shown promising results in vitro, in animal models, and in early clinical trials, their widespread application is still hindered by issues such as synthetic quality control, large-scale production, standardization, long-term safety assessment, personalized treatment protocols, and integrated platforms for multifunctional diagnostics and therapeutics. Despite these challenges, the synergistic development of materials science and medical engineering holds the potential to drive significant advancements in the application of INPs in colorectal cancer, offering patients more accurate, safe, and efficient treatment options.

## Figures and Tables

**Table 1:** Comprehensive overview of past and ongoing clinical trials in colorectal cancer.

Status	Initial Time	Categories	Aim	Clinical Trial/Reference
Completed	2010	Fe_3_O_4_ nanoparticles	Thermal ablation	[33]
Completed	2015	USPIO contrast agent (ferumoxtran-10)	Lymph node metastases	NCT02751606 [40]
Completed	2017	Carbon nanoparticles	Tumor localization and lymph node mapping	NCT03350945
Completed	2019	Hafnium oxide nanoparticles	Radio therapy	NCT02379845 [34]
Completed	2021	Carbon nanoparticle	Lymph node tracer	NCT04759820
Completed	2022	Carbon nanoparticles	Lymph node tracing and surgery guiding	NCT06783985
Ongoing	2023	Carbon nanoparticle-loaded iron [CNSI-Fe (II)]	Ferroptosis	NCT06048367 [35,36,37]
Ongoing	2024	Ultrasmall superparamagnetic iron oxide (USPIO)	Evaluation of lymph node metastasis and staging	NCT06693375 [38,39]

**Table 2:** Summary of the physical properties of different types of INPs and their diagnostic and therapeutic applications in colorectal cancer.

Categories	Physical Property	Diagnosis in Colorectal Cancer	Treatment of Colorectal Cancer
Metal Nanomaterials	Optical properties	CT [113,114,115,116,117], photoacoustic imaging [118,119,120], fluorescence imaging [117,121,122,123]	Photothermal [118,119,121,124,125,126], photodynamic [127,128,129,130]
	Magnetic properties	MRI [131]	Magnetothermal [123]
	Acoustical property		Acoustic force [120,132]
	High specific surface area and surface effect, biocompatible		Targeted drug delivery [133,134,135]
Metal Oxide Nanomaterials	Optical properties	CT [136], photoacoustic imaging [137,138,139], fluorescence imaging [123]	Photothermal [123,137,139], photodynamic [136]
	Magnetic properties	MRI [114,136,138]	Magnetothermal [140,141,142]
	Catalytic properties		Nano-enzymatic activity [143]
	High specific surface area and surface effect, biocompatible		Targeted drug delivery [144,145,146]
Quantum Dots	Optical properties	Fluorescence imaging [147,148,149,150,151], molecular probes [152]	Photothermal [119,153]
	High specific surface area and surface effect, biocompatible		Targeted drug delivery [151,153,154,155,156,157,158]
Carbon-based Nanomaterials	Optical properties	Photoacoustic imaging [124]	Photothermal [124,159], photodynamic [160,161,162]
	High specific surface area and surface effect, biocompatible		Targeted drug delivery [158,160,162,163,164,165,166,167]
Magnetic Nanomaterials	Optical properties		Photothermal [168,169,170,171] Photodynamic [172,173]
	Magnetic properties	MRI [174,175,176,177]	Magnetothermal [178,179,180,181,182]
	Catalytic properties		Nano-enzymatic activity [183]
	High specific surface area and surface effect, biocompatible		Targeted drug delivery [174,184,185,186]
Rare-earth Nanomaterials	Optical properties	PET-CT [187,188], fluorescence imaging [189,190]	Photothermal [191]
	Magnetic properties	MRI [192]	
	Catalytic properties		Nano-enzymatic activity [191,193]
	High specific surface area and surface effect, biocompatible		Targeted drug delivery [192,194,195]
